# Tribute to the Medical Profession

**Published:** 1849-08

**Authors:** 


*Tribute to the Medical Profession.*—

We find the following tribute to the character of the medical profession, in the columns of the *Daily Express*, of this city, the prefatory remarks, referring to the Physicians of Buffalo, being by the Editor of that print. While we trust and believe, that the fortitude and disinterestedness evinced by our profession in periods of public calamity from the prevalence of a pestilential disease, proceed from higher aims and motives than the approbation of mankind, it is doubtless a source of gratification and encouragement to know that their labors, by a portion of the community, at least, are more than appreciated.

*Well Merited Praise.*—The Richmond Republican pays the following tribute, which we doubt not is deserved, to the physicians of that city, for their conduct during the prevalence of the cholera. The same language is applicable to the physicians of our own city, who, as a body, take rank in learning, skill, and self-devotedness, with those of any other city in the count y:

*Physicians.*—We cannot observe, without the strongest admiration, the conduct of the physicians of Richmond during the present epidemic, and we cannot forbear from publicly expressing that admiration, however feeble may be the language in which we endeavor to convey our feeling. We do not claim for our physicians greater devotion than is manifested by their profession in other cities, but we claim for the profession here, and every where, that it is one of the noblest professions on the face of the earth. It is only at times like these that we fully realize the excellence of these true-hearted sons of science, these heroic men, in comparison with whose calm courage the fiery valor of the soldier shrinks into utter insignificance. A period of ordinary health is to the physician like a time of peace to the soldier, but the visitation of the epidemic, is the war in which he goes forth to the front of the battle, and to the struggle with death, that he may save the lives of others, and perhaps perish himself in saving them. Yet we hear many say that physicians are paid for their services! And are not all other physicians paid? Are not the soldier and the sailor paid? Were not Jackson and Taylor, Perry and Decatur, paid for their services? No. A grateful country placed a wreath of immortal glory upon their brows, far more valuable than gold; a wreath which the faithful physician deserves equally with a Napoleon or Wellington.

Look at the conduct of our physicians here. See them, old and young, pressing forward like a band of chivalrous brothers to the relief of suffering humanity. There is no hovel so poor, so loathsome, so reeking with the foul breath of the pestilence, in which those messengers of mercy have not been found standing by the bedside of the most miserable and destitute wretch in the community, no matter what his color, and exhausting all the resources of medical skill for his relief. In cases like these there could have been no remuneration. None was expected. But that mattered not. Life was at stake, and as rapidly as others would fly from the danger, have our physicians hurried to it, to save their fellow men.

Listen in the dark night, and at all hours, you hear them driving by in hot haste to the help of some victim of the pestilence. Sleep is a rare thing to them. Sometimes they obtain two or three hours of rest, and then are roused again to their exhausting duties. Sometimes night after night passes without their obtaining a minute of repose. Contagion they laugh at. The fatigue that exposes them to the epidemic they scorn. With a glorious enthusiasm they devote themselves to the benefit of their fellow men. We have heard of deeds of generosity on the part of physicians, we have known facts illustrating their nobility of nature, which we cannot publish, but we could not forget, if we should live a hundred centuries, deeds which must surely receive the approbation of the Great Physician of souls, and be remembered at that decisive hour when the declaration, "For I was hungered, and ye gave me meat: I was thirsty, and ye gave me drink: I was a stranger, and ye took me in: naked, and ye clothed me: I was sick, and ye visited me," shall send unspeakable joy to every pure and benevolent heart.

"Honor to whom honor is due." This is a feeble tribute, but it expresses in faint terms what thousands strongly feel.

